# Is COVID-19 Fatality Rate Associated with Malaria Endemicity?

**DOI:** 10.15190/d.2020.17

**Published:** 2020-12-11

**Authors:** Abdul Rehman Arshad, Imtiaz Bashir, Farhat Ijaz, Nicholas Loh, Suraj Shukla, Ubaid Ur Rehman, Rana Khurram Aftab

**Affiliations:** ^1^CMH Lahore Medical College and Institute of Dentistry (NUMS), Lahore, Pakistan; ^2^Flinders University, College of Medicine and Public Health. Adelaide, SA, Australia; ^3^Punjab Institute of Cardiology, Lahore, Pakistan

**Keywords:** Malaria, Plasmodium, COVID-19, SARS-CoV-2, endemic, case fatality rate, ACE2, IFN-γ, CD147.

## Abstract

COVID-19 (coronavirus disease 2019) is a disease caused by the coronavirus SARS-CoV-2 (severe acute respiratory syndrome coronavirus 2). COVID-19 has yielded many reported complications and unusual observations. In this article, we have reviewed one such observation: an association between malaria endemicity and reduced reported COVID-19 fatality. Malaria-endemic regions have a significantly lower reported COVID-19 fatality rate as compared to regions where malaria is non-endemic. Statistical analyses show that there is a strong negative correlation between the reported SARS-CoV-2 fatality and endemicity of malaria. In this review, we have discussed the potential role of CD-147, and potential malaria-induced immunity and polymorphisms in COVID-19 patients. Noteworthy, the results may also be due to underreported cases or due to the economic, political, and environmental differences between the malaria endemic and non-endemic countries. The study of this potential relationship might be of great help in COVID-19 therapy and prevention.

## Summary

*1. Introduction*

*2. Materials and Methods*

*3. Proposed Relationship Between Malaria and COVID-19*

- *3.1.* *Distribution of COVID-19 and malaria*

- *3.2. Effectiveness of hydroxychloroquine in COVID-19*

- *3.3. Negative correlation between malaria and COVID-19 burden and fatality *

*4. Proposed Mechanisms to Explain this Epidemiological Paradox*

- *4.1. Interferon-γ might play a role*

- *4.2. Neutralizing antibodies from malarial infection might be helpful against COVID-19 infection*

- *4.3. CD-147 receptor, the common entry point for malarial plasmodium and coronavirus might be helpful against COVID-19 infection*

- *4.4. Malaria might have induced polymorphism in ACE-II gene*

*5. Limitations and Alternative Explanations*

*6. Conclusion*

## 1. Introduction

COVID-19 (coronavirus disease 2019) is a disease caused by the coronavirus SARS-CoV-2 (severe acute respiratory syndrome coronavirus 2). The ongoing pandemic has exhibited unusual features and made many headlines. For example, hydroxychloroquine had been initially touted as a potentially effective treatment against COVID-19. However, US Food and Drug Administration (US FDA) approval for hydroxychloroquine use as emergency and compassionate treatment was soon withdrawn.

Moreover, widely reported complications and anomalous presentations began to emerge, such as the case of a sinus venous thrombosis in a 59-year-old man^1^. There is certainly no paucity of unusual COVID-19-associated findings that can be found in literature. We would like to highlight yet another unusual finding: the association between malaria endemicity and a reduced reported COVID-19 fatality rate.

As global COVID-19 cases and deaths rise, we have observed great geographical discrepancy in the number of cases and deaths. The United States and various European countries have reported a relatively large number of cases and deaths. However, the majority of countries in Africa, South America, and in the subcontinent (South Asia) have comparatively reported substantially fewer number of cases and deaths^2^. In fact, several African countries (e.g. Nigeria, Ghana, Ivory Coast and Kenya) report fewer than five deaths/million of population; this is in contrast to European countries where on average, there are more than five hundred deaths/million of population^2^. Malaria is a disease that dates as far back as humans. Its longevity can be attributed in part to some of the characteristics of the malarial parasite that are thought to have co-evolved in tandem with humans^3,4^. Despite malaria’s longevity and its present endemicity in several geographical regions worldwide, an effective vaccine for the disease has yet to be developed^5^.

There is a need to bridge the knowledge gaps established by attempting to produce a plausible explanation for the low COVID-19 fatality in malaria-endemic countries.

## 2. Materials and Methods

An organized review of literature relevant to the topic was developed, considering original articles, and current research and literature. Data for statistical analysis was obtained from the World Health Organization (WHO)’s COVID-19 case reports. The key terminologies used for the search were: malaria and COVID, malaria endemicity and COVID fatality, COVID-19. Search engines used were Google Scholar, PubMed, MEDLINE, and EBSCOHost. A total of 10 articles were selected based on their possible relevance for the topic of interest.

## 3. Relationship Between Malaria and COVID-19

### 3.1. Distribution of COVID-19 and malaria

**Figure 1:**
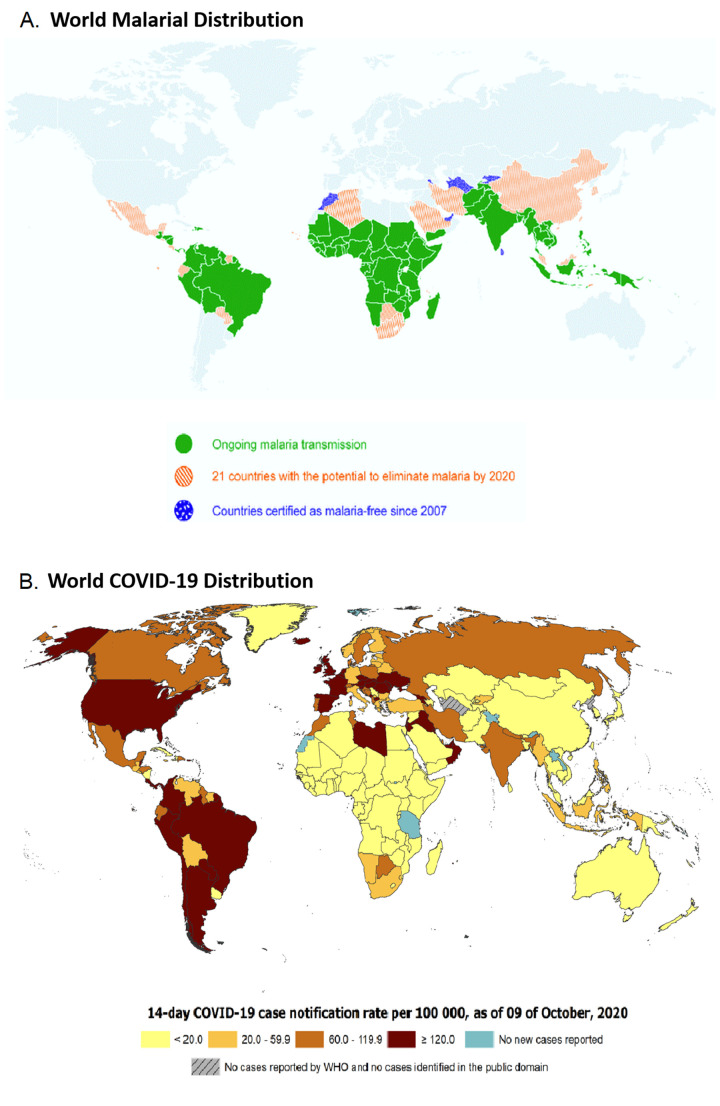
Comparative Worldwide Distribution of Malaria and COVID-19 **A.** Worldwide distribution of malaria; reproduced from Rabinovich RN et al.^6^, with permission; **B.** Worldwide distribution of COVID-19; reproduced from reference^7^, with permission.

Figure 1^6,7^ contains the world map distribution for malaria and COVID-19. A straight side by side look at the two graphs shows the stark contrast between the two diseases. The less spread can be appreciated in malaria non-endemic countries. It points out that there might be an association between the higher rates of malaria and lesser infectivity of COVID-19.

### 3.2. Effectiveness of hydroxychloroquine in COVID-19

The relationship between malaria and coronaviruses was first pointed out back in 2005, when the antimalarial drug chloroquine was shown to be highly effective against the spread of SARS-CoV-1, even at prophylactic doses^8^. 15 years later, in the initial stages of the COVID-19 pandemic, chloroquine use was proposed to be able to reduce COVID-19 fatality^9^. Unfortunately, it was discovered that COVID-19 patients taking chloroquine were at a higher risk of death and cardiac issues^10^. Since comorbidities have already been suggested to be one of the major risk factors for fatality in COVID-19 patients^11^, WHO was essentially compelled by a peer-reviewed paper to retract their statement regarding hydroxychloroquine and halt hydroxychloroquine clinical trials. However, since then, the same paper has been retracted over reservations on the accuracy of the data. This so-called efficacy of hydroxychloroquine also points out some correlation between malaria and COVID-19.

### 3.3. Proposed Negative Correlation between Malaria and COVID-19 Burden and Fatality

**Figure 2:**
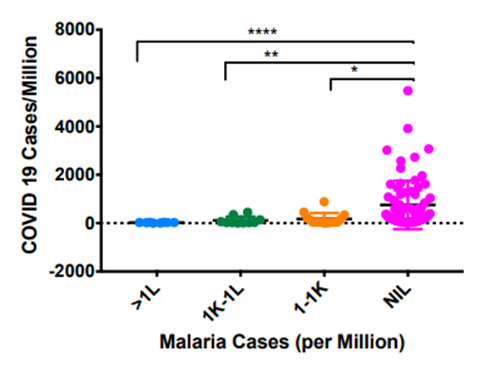
Relation Between Malaria Cases/Million and COVID Cases/Million Reproduced from Muneer A et al.^12^, with permission.

**Figure 3:**
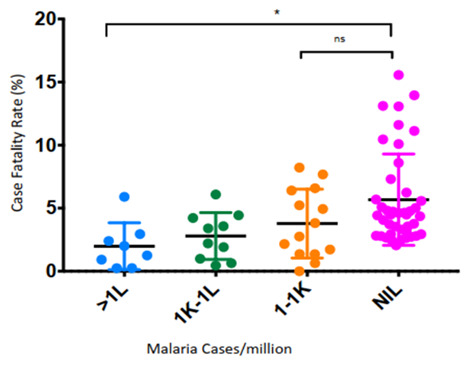
Relation Between Malaria Cases/Million and Case Fatality Rate (%) Reproduced from Muneer A et al.^12^, with permission.

A study performed by A Munir et al.^12^ describes an inverse correlation between indices of malaria in 2018 and COVID-19 infections (r = -0.15, p = 0.02). They analyzed the data of 108 countries labelling them according to malaria burden as: (i) No malaria, (ii) 1-1000, (iii) 1000 – 100 thousand, (iv) > 100 thousand malaria cases / million in 2018. Countries in the first category had highest number of COVID-19 cases, while the fourth category had lowest number of these cases. The share of these categories to total number of COVID-19 cases were 94%, 2.5%, 3.3%, and 0.2% respectively. Furthermore, fatality rates of COVID-19 followed a similar pattern: 6.63%, 3.88%, 4.63%, and 2.58% respectively (Figure 2 and Figure 3). Similar correlations have been discussed in the study done by Napoli et al.^13^.

Table 1 shows a comparison of the current data on COVID distribution and fatality rate of the top 20 most affected countries by COVID-19, based on data from WHO’s COVID-19 case reports. Countries have been labelled as malaria endemic or non-endemic, on the basis of WHO’s malaria reports.

**Table 1:** Current COVID-19 status of Malaria endemic and non-endemic countries Spearman’s correlation gives a negative correlation between malaria endemicity and death rate (r = -.434, p < 0.05); https://www.worldometers.info/coronavirus/

Name of the country	Number of reported cases	Number of reported deaths	Malaria status
Italy	344,000	36,111	Non-Endemic
Iran	492,000	28,098	Endemic
Mexico	810,000	83,497	Non-Endemic
Pakistan	318,000	6558	Endemic
France	692,000	32,583	Non-Endemic
Saudi Arabia	339,000	4996	Endemic
Canada	179,000	9558	Non-Endemic
Bangladesh	376,000	5477	Endemic
Spain	861,000	32,929	Non-Endemic
Peru	839,000	33098	Endemic
United Kingdom	576,000	42679	Non-Endemic
Chile	478,000	13,220	Endemic
Belgium	149,000	10,151	Non-Endemic
Qatar	128,000	219	Endemic
Germany	320,000	9599	Non-Endemic
South Africa	688,000	17,547	Endemic
USA	7700000	214,000	Non-Endemic
India	6980000	107,000	Endemic
Russia	1270000	22257	Non-Endemic
China	91170	4512	Endemic

## 4. Proposed Mechanisms to explain this Epidemiological Paradox

Several hypotheses have been made to explain the role of malaria in altering the pathogenicity of COVID-19 infection.

### 4.1. Interferon-γ might play a role

The role of IFN- γ in COVID-19 is described by Channappanavar et al.^14^. Angiotensin II primarily mediates through its pro-inflammatory properties an increase in the pro-inflammatory cytokines, such as IFN-γ, IL-6, IL-10^15^, as well as IL1B, IP10 and MCP1^16^, released in several tissue sites.

IFN-γ has been touted as one of the quarterbacks of the immune system’s response to the virus. Elevated levels of IFN-γ in the beginning of the response to the viral infection have been associated with attenuated disease progression and decreased fatality. However, elevated levels of IFN-γ in the later stages of the disease have been associated with a worse prognosis^17,18^. Hence, the importance of IFN-γ may not rest in the quantity of IFN-γ released per se, but rather in the timing of the IFN-γ response. In this manner, the ability of the patient to launch a timely and well-regulated IFN-γ response seems to be essential for a better prognosis in a COVID-19 infection.

A similar phenomenon has also been observed in the patients infected with malaria. African children who were able to mount a quick, early, and robust IFN-γ response were only associated with mild incidences of malaria, whereas those with severe malaria were actually found to have much higher systemic levels of IFN-γ^19^. Artavanis-Tsakonas et al. in their paper hypothesize that the reason why children who are undergoing their first infection with malaria are less likely to develop cerebral malaria, compared to older children who have already been exposed a couple of times is because, in the latter group, cross-reactive primed T-cells are still being developed, and in the midst of the ‘practice’, they produce copious amounts of IFN-γ, as compared to their younger counterparts^20^. However, with constant re-exposure, as would occur in the setting of a malaria-endemic region, eventually the immune response has matured enough to produce IFN-γ in a more efficient manner, whilst also damping the pro-inflammatory cytokine cascade, thereby priming the innate system and T-cells^20^. To summarize, children on their first exposure to malaria are unable to produce sufficient IFN-γ. However, they have a better prognosis than children who have already been exposed once or twice and produce copious amounts of IFN-γ. People infected multiple times are able to launch a more efficient, timely, and well-regulated immune response and they are associated with the best prognosis. This demonstrates the same phenomenon observed in COVID-19 (as discussed above); it is not the amount of IFN-γ produced during an infection, but rather the timing, efficiency, and regulation of production of IFN-γ that is associated with survival.

### 4.2. Neutralizing antibodies from malarial infection might be helpful against COVID-19 infection

Randell and Alexander^21^ hypothesized that there may be natural immunity against COVID-19 infection in the populations that are constantly exposed to malarial infections. Studies show that there is a protection against reinfection provided by interferon gamma, CD8+ T cells and nitric oxide. Repeated exposure to malarial infections also induces the development of persisting neutralizing antibodies that neutralize a broad profile of merozoite antigens^22,23^ and are also noted to have effects against coronaviruses, including SARS-CoV-2^23^.

### 4.3. CD-147 receptor, the common entry point for malarial plasmodium and coronavirus might be helpful against COVID-19 infection

**Figure 4:**
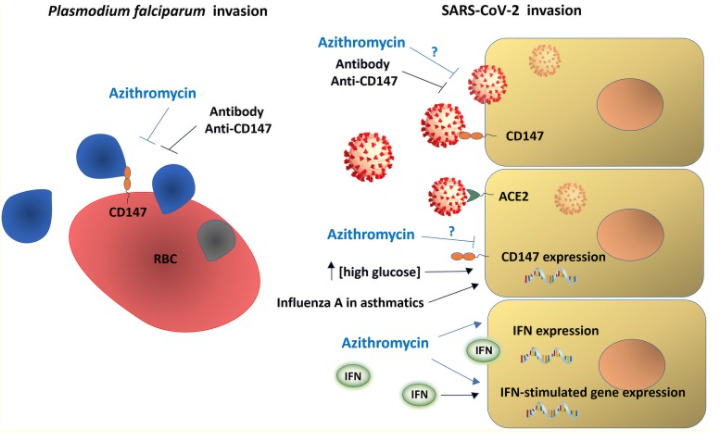
CD-147 as a common receptor for plasmodium and SARS-2-coronavirus; potential action of azithromycin in COVID-19 R**eproduced from Ulrich H et al.^24^, with permission.**

Ulrich et al^24^ described the effects of azithromycin in the treatment of COVID-19 and explained the role of CD-147 in the pathogenesis of the disease. CD-147 (emmperin) is the common target receptor for both malaria and COVID-19. CD-147 is expressed on several immune cells where it causes induction of chemotactic cytokines (TNF-alpha, IL-10, IL-6), causes MMP2, IL-9 induction, production of IFN-γ (IL-18), T-cell activation, proliferation, invasion, adhesion and energy activation^25^. Multiple studies have found that the release of interferons by lymphocytes represents a normal immune response to infection with any of the multiple strains of malaria, and that these interferons have both in vivo and in vitro effects against coronaviruses responsible for SARS, MERS, and COVID-19^22,26^. Malaria-induced natural immunity to COVID-19 infection might be triggered through this common route, i.e. CD-147. This is further backed up by the role of CD-147 blockers in COVID-19 infection (Figure 4).

### 4.4. Malaria might have induced polymorphism in ACE-II gene

The wide-varying of clinical manifestations of malaria have been attributed to the ability of malaria to induce polymorphisms in genes that encode for the isozymes of angiotensin-converting enzyme (ACE): angiotensin-converting enzyme 1 (ACE1) and angiotensin-converting enzyme 2 (ACE2)^27^. ACE2 has been involved as a binding site for the SARS-CoV-1 spike (S) protein^28^; specifically, the S1 domain, where a change in degree of binding site of SARS-CoV-1 to ACE2 is associated with genetic polymorphism^28^. Due to the genetic similarity between SARS-CoV-1 and SARS-CoV-2, this may have implications for individual susceptibility to SARS-CoV-2 infection, and thus, possibly COVID-19 disease prognosis^28,29^. In fact, in the same experiment, S proteins obtained from 2003-2004 pandemic strain bound markedly less efficiently than the S proteins isolated from the 2002-2003 pandemic strain, which was actually a more deadly strain of the virus. The notion of a ‘different strain’ and genetic polymorphism in ACE2 has been suggested to be responsible for the initially high fatality rate reported in Iran^30^, and Italy^31^, as well as in conferring a protective role against coronavirus^13^.

## 5. Limitations and Alternative Explanations

Due to the lack of proper evidential data, there may be errors in the reported numbers, due to incorrect reporting of the actual number of cases. An incorrect figure might be due to the varying amount and capacity of testing of different countries. As all the non-endemic countries are essentially more developed than the endemic ones and have a different form of organization and leadership, this might lead to understatement or overstatement of the actual number. There is no way to completely remove these errors. However, to ensure the uniformity of the collected information, all the facts and figures were taken from the WHO’s case reports, which is the most employed way of obtaining this data so far.

## 6. Conclusion

Malaria-endemic regions have a significantly lower COVID-19 fatality rate as compared to regions where malaria is non-endemic. Statistical analyses indicate a strong negative correlation between SARS-CoV-2 and endemicity of malaria, although some errors in COVID-19 reporting can’t be excluded. This might be due to malaria induced natural immunity against COVID-19 infection in the countries where malaria is endemic. CD-147, the common receptor for malaria and COVID-19 might serve a role in this immunity. Furthermore, malaria is known for inducing gene polymorphisms, so it might have produced some sort of polymorphism that might be playing a protective role against COVID-19 pathogenesis. The study of this relationship might be helpful to find out a treatment for COVID-19.
